# A Feasibility Study of a Music Enrichment Program on Relative Reinforcing Value of Food and Home Environmental Enrichment among Families of Low Socioeconomic Status

**DOI:** 10.3390/children11101229

**Published:** 2024-10-11

**Authors:** Kai-Ling Kong, Amy R. Smith, Brenda Salley, Deanna Hanson-Abromeit, Hideko Engel, Catherine A. Serwatka

**Affiliations:** 1 Baby Health Behavior Lab, Division of Health Services and Outcomes Research, Children’s Mercy Research Institute, Children’s Mercy Hospital, Kansas City, MO 64108, USA; amy.smith.1@vumc.org (A.R.S.); hmengel2@wisc.edu (H.E.); cserwatka@cmh.edu (C.A.S.); 2 Department of Pediatrics, University of Missouri, Kansas City, MO 64108, USA; 3 Center for Children’s Healthy Lifestyles and Nutrition, Children’s Mercy Hospital, Kansas City, MO 64108, USA; 4 Department of Pediatrics, University of Kansas Medical Center, Kansas City, KS 66103, USA; bsalley@kumc.edu; 5 Department of Pediatrics, Children’s Mercy Hospital, Kansas City, MO 64108, USA; 6 Department of Music Education & Music Therapy, School of Music, University of Kansas, Lawrence, KS 66045, USA; dhansonabromeit@ku.edu

**Keywords:** relative reinforcement, infant obesity, low socioeconomic status, environmental enrichment, music enrichment program, home language environment, quality of parent–infant interactions

## Abstract

Background: Emerging evidence suggests that low socioeconomic status (SES) home environments may play a role by promoting excess energy intake through a lack of access to non-food reinforcers. Because of the deleterious effects of SES-related disparities on child health and development, feasible and culturally acceptable interventions are urgently needed. Community-based music enrichment programs may be an ideal intervention strategy. Methods: In collaboration with a local non-profit organization and music studio, we conducted a pilot randomized controlled trial to assess the effects of a music enrichment program versus a play date control in a group of 9–24-month-old healthy infants (N = 16). The study was conducted in accordance with the Declaration of Helsinki and approved by the Institutional Review Board of Children’s Mercy Hospital Kansas City. This study is registered with clinicaltrials.gov (NCT05868811). Results: Overall, we found some intervention effects on the relative reinforcing value of food (RRV_food_) and the home environmental enrichment measures (i.e., increased music use at home and the home language environment). Our intervention demonstrated large effects on the increased use of music at home. We did not find significant group differences in the RRV_food_ and home language environment, but some of the effect sizes were medium-to-large. Results also suggest that our intervention is feasible and acceptable. Parent feedback indicated that the intervention was well-liked and that the steps we took to help reduce barriers worked. Conclusions: Music enrichment programs may be a high-impact, low-cost strategy to address socioeconomic disparities.

## 1. Introduction

Emerging evidence suggests that low socioeconomic status (SES) home environments may play a role by promoting excess energy intake through a lack of access to non-food reinforcers, which may lead to an imbalance of food versus non-food reinforcement [1]. One way to study eating in the context of multiple behavioral alternatives is to measure the relative reinforcing value of food (RRV_food_), specifically studying how hard individuals will work for food versus a non-food alternative. High RRV_food_ has been associated with lower education levels and household income [1], higher energy intake, and an increased prevalence of obesity [2]. A recent study showed that exposure to certain familial traits during infancy (e.g., maternal sensitivity, cognitively stimulating activities) lowered the chances of obesity and buffered the negative impact of concurrent familial risk (e.g., poverty, single-parent household) [3]. Further, there is robust evidence that shows children from low-SES households whose parents create enriching home environments that include positive parenting and access to cognitively stimulating activities, experience enhanced language development, perform better academically, have greater self-regulation, and have a reduced likelihood of obesity relative to matched-on counterparts [4,5,6,7,8,9].

Children grow up in homes that differ vastly in their environmental enrichment. The concept of environmental enrichment has been extensively studied by many developmental scientists in relation to language and cognitive outcomes. For example, music enrichment programs have been shown to improve receptive communications skills [10] and use of gestures, an indicator of early communicative ability [11]. Book sharing/reading programs can enhance the early language environment and subsequent academic success [12]. Enriched environments can reduce sensitivity to rewards [13,14], and maladaptive choices [15]. Early non-food home environments that promote comfort and pleasurable behaviors as alternatives to eating may mitigate young children’s food-seeking behavior and thus alter the obesity trajectory. A few prospective studies demonstrate that increased access to cognitively stimulating activities (e.g., access to musical instruments, toys, reading materials, and going to shows and museums) is associated with lower levels of weight gain [16,17]. Moreover, observational data suggest that having positive relationships with parents during infancy [18], mothers who are highly sensitive and responsive [19], and parents with a high degree of warmth [17] are protective factors against childhood obesity.

Because of the deleterious effects of SES-related disparities on child health and development, feasible and culturally acceptable interventions to help enrich the home environment of families from low-SES backgrounds are urgently needed. Thus far, obesity prevention research has primarily focused on one’s home food environment. Many of these interventions have yielded limited success in childhood obesity prevention, especially those targeting low-income, marginalized racial/ethnic groups [20,21]. Outside of obesity research, positive parenting interventions have been used to improve cognitive and socio-emotional outcomes in young children, especially among children from disadvantaged homes. More recently, obesity researchers have leveraged the knowledge attained from this line of research by promoting responsive parenting, primarily in the feeding or food domain of an infant’s home environment. Despite initial success in preventing weight gain, many of these responsive feeding interventions have not yielded long-term effects (≥3 yr) [22,23,24,25,26]. On the other hand, evidence is emerging to show that parenting interventions *not* targeting weight and feeding/home food environment are having a positive impact on children’s long-term health outcomes [27,28,29,30]. A recently published systematic review shows that positive and responsive parenting can positively impact a child’s risk of obesity for up to 10 years post-intervention, including many cohorts of diverse, low-income families [31]. Perhaps these programs, in part, enrich the home environment of those children and indirectly benefit their physical health.

Community-based music enrichment programs such as Music Together^®^ may be an ideal intervention strategy to address this disparity and narrow the gaps. Observational research with typically developing children has shown that parent–child musical interactions capture infants’ attention and synchronize the dyad’s arousal and affect, more so than during non-musical interactions [32,33]. Participating in a music enrichment program has been shown to improve parent–child interactions and parenting behavior and reduce parent stress [10,34]. More recently, parent–infant dyads who participated in a music enrichment program for one year had a greater increase in conversational turns compared to dyads in a play date control group [35]. Music enrichment programs provide a rich context (e.g., singing, dancing, playing musical instruments) for high-quality parent–child interaction, which is related to enhanced health and developmental outcomes. Music enrichment programs are valued for their broad developmental benefits for all children, regardless of their individual circumstances or risk factors, which may help break down stigmas toward intervention among low-SES families. Additionally, music enjoyment is cross-cultural, with parents across all ethnicities and demographics singing to their infants [36]. Music enrichment programs are typically embedded in many middle- to high-income communities and are associated with high levels of parent engagement and attendance [37]. However, these programs generally do not reach children from low-SES families due to location, financial constraints, or cultural norms.

Our lab was the first to evaluate the possibility of music enrichment programs to prevent obesity [blinded reference]. To date, our work has been conducted primarily among highly educated and mid–upper-income families. Thus, the focus of the current study is to gather crucial information on cultural acceptance and feasibility that will inform tailoring of music enrichment programs for use with families from low-SES households. Once we have an understanding of the acceptability and feasibility of music enrichment programs in low-SES households, we can begin to adapt obesity prevention interventions to communities that will benefit the most. In collaboration with a local non-profit organization and music studio that serve primarily low-SES families, we conducted a pilot randomized controlled trial (RCT) to assess the effects of a music enrichment program versus play date control in a group of 9–24-month-old healthy infants (N = 16). We examined the effects of enhancing alternatives to eating using a music enrichment program on the RRV_food_ and home environmental enrichment measures. It was expected that the music enrichment program would increase the reinforcing value of music, which would in turn decrease the overall RRV_food_ of participants in the music group. Thus, we hypothesized that, following our intervention, infants in the music group would have a lower RRV_food_ and more enriched home environment (i.e., increased music use at home and a higher quality home language environment) compared to infants in the control group. We also wanted to determine the acceptability and feasibility of a music enrichment program as a multi-target intervention among families from low-SES households.

## 2. Methods

### 2.1. Participants

**Figure 1:**
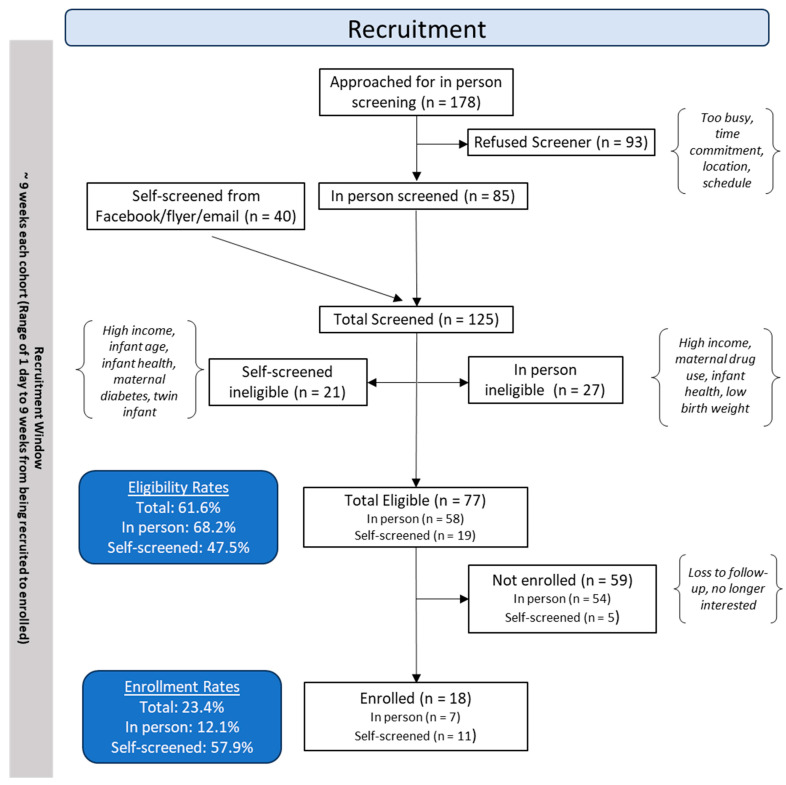
Full recruitment and enrollment flow chart.

**Figure 2:**
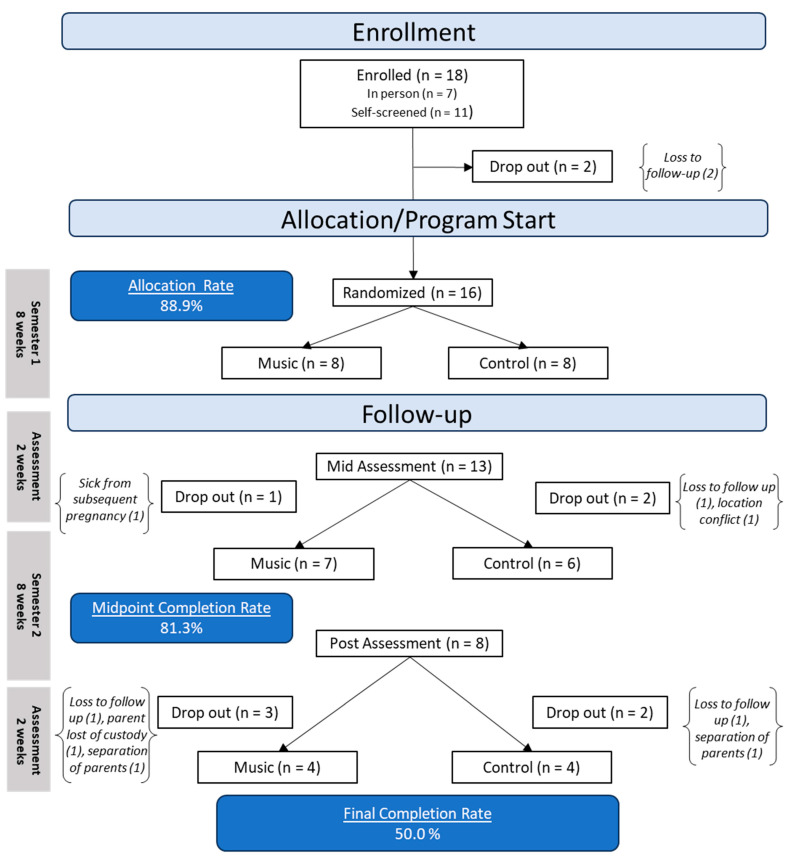
Full randomization and retention flow chart.

Families with children aged 9–24 months were recruited from December of 2022 to September of 2023 using posted flyers, Facebook, and in-person recruitment at a hospital-affiliated primary care clinic. Parent–child dyads were excluded from participation if the infant was preterm (<37 weeks of gestation) or had known developmental delays or disabilities according to the maternal report; if the maternal age was <18 years at the time of pregnancy; if any maternal smoking or illicit substance use during pregnancy was reported; if maternal alcohol use of >4 alcoholic drinks on a single occasion or an average of >1 alcoholic drink per day during pregnancy was reported; if the pregnancy was a high-risk pregnancy (e.g., occurrence of placenta abruption, pre-eclampsia, gestational diabetes); or if the dyad did not speak English. We enrolled a total of 18 families; we lost contact with two families prior to randomization and the start of the program, so they were excluded. Thus, we randomized *n* = 16 families into two groups: the music enrichment program or the play date control. Figure 1 and Figure 2 provide a full participant flowchart.

#### Socioeconomic Status (SES)

Sociodemographic factors were assessed using a modified MacArthur Questionnaire upon enrollment [38,39,40]. Questions included years of education, single-parenthood, race/ethnicity, individual and household income levels, and household size. With the aim of recruiting a low-SES sample, we only recruited participants who were using the Medicaid program (used as a proxy for income since some families did not respond to questions related to income). Using the same criteria as Base Academy of Music, a local music studio that serves diverse, low-income families in the Kansas City Metro area, families were eligible if they qualified for the Special Supplemental Nutrition Program for Women, Infants, and Children (WIC) (household income < 185% of the Federal Poverty Level).

### 2.2. Study Design

We conducted a randomized, controlled pilot trial to assess the effects of a non-food, music enrichment program (Music Together^®^; music group) compared with an attention play date program (control group) on infant health and development as well as to assess feasibility and acceptability of the study. Primary outcome measures included motivation for food (RRV_food_), along with music and language environments in the home. The program consisted of two semesters of 8 weekly intervention sessions. Additionally, baseline/pre-intervention, mid-intervention, and post-intervention assessments were completed. The study is a mixed study design with the group as the between-subject factor and baseline, mid-, and post-intervention assessments as the within-subject factor. Eligible infants were stratified by sex and age, then we randomly assigned one subject from each pair to the intervention or control groups, music (*n* = 8) or control (*n* = 8). This was performed to minimize selection bias. One caregiver served as the participating parent who attended most of the classes and assessment visits with their infant (mother, *n* = 15; father, *n* = 1).

### 2.3. Procedures

The study procedures were approved by the Institutional Review Board at [BLINDED]. Interested parents were screened via an online questionnaire or in-person interview at a hospital-affiliated primary care clinic. Eligible families were scheduled for two baseline assessment visits: a home visit (~45 min) followed by a lab visit (~45 min). When researchers arrived at the home, parents were given a brief description of study protocols and completed a consent form for their participation and the infant’s participation. During the visit, parents filled out study questionnaires. Parents were then provided with instructions to record two days of the infant’s natural language environment using the Language Environment Analysis (LENA) devices. Before leaving their home, parents scheduled their second lab assessment visit. This visit was scheduled during a time when the parent felt the infant would be awake, alert, and willing to do the food/non-food reinforcement task. Parents were instructed to avoid feeding their child one hour prior to the visit and to provide the infant’s favorite solid food for the food portion of the task. Upon the family’s arrival to the lab, researchers interacted with the infants, establishing rapport by using toys and reading books. While infants became familiar with the researchers, parents completed study questionnaires. This orientation period lasted 5–10 min, until the infant had acclimated to their surroundings, as confirmed by the parent. Then, the child was placed in a highchair next to the parent to avoid separation anxiety and stranger anxiety. Parents were informed not to interact with the child during any of the research tasks. After the food/non-food reinforcement task, research staff measured the height and weight of the parent and infant.

After completing both baseline assessment appointments, families were randomly assigned to the music or control group. Families completed follow-up assessments using the same procedure after their first semester (8-week) and second semester (16-week). When possible, the same research staff ran appointments so that families remained familiar with the staff, especially the researcher who delivered the reinforcers to the infants. Lastly, we sent out a parent satisfaction survey via email to all enrolled families to assess the acceptability of the intervention.

### 2.4. Intervention

The intervention took place at a community location, [Blinded], which primarily serves families of low-SES backgrounds. The music enrichment and play date program were scheduled one evening per week at the same time. Participants in both groups attended the weekly sessions for two 8-week semesters. The programs were administered in a group format, and families were given the opportunity to attend one make-up session at the end of each semester if they missed any sessions. To ease the burden on families, dinner was served each week before the start of the program, and childcare was provided if the participating parent brought siblings or other family members to the sessions. All participants were offered free transportation through the Lyft service to attend classes. Lastly, families were compensated with USD 20 each week after attending the class to offset any costs that might have incurred due to their participation in the program.

#### 2.4.1. Music Enrichment Program

Families in the music enrichment group attended the Music Together^®^ program. This program introduced families to the pleasure of making music rather than passively receiving music from devices/screens. This program provided a rich variety of musical activities, which encourage children and parents to participate at their own level in singing, moving, listening, or exploring musical instruments. A board-certified music therapist with 10 years’ experience working with young children and trained in the Music Together^®^ curriculum facilitated all the group classes. Music Together^®^ training included 40 h of course work covering key aspects of child musical development, facilitation, and curriculum. Participating parents and infants attended 45 min classes as a group. Besides attending classes, parents were encouraged to listen to music and sing together with their infants at home during everyday activities such as bath time, mealtime, and bedtime using the music and instructional song book provided on the first day of the program.

#### 2.4.2. Play Date Control Program

Families in the control group attended 45 min play date group sessions at the same intervals, location, and time as the music group. Before the arrival of parents and infants, play stations were set up, and the room was childproofed. The research staff provided the parents and infants with a variety of age-appropriate toys (excluding musical toys) to play with and enjoy. Parents were encouraged to interact with other parents and infants during the 45 min play time. A research staff member with experience running play dates was present during each play date session to facilitate the play. The staff member modeled playing with the infants and encouraged parents to engage in playing, but they did not discuss any developmental milestones or provide any play instructions to parents. Besides attending play dates, families were given toys to play with at home. Parents were told it was a way for them to spend time bonding with their children. Each family was provided with one new toy to play with at home each semester (stacking cups and a zoo animal puzzle). Parents were encouraged to play with their child at home during everyday activities such as bath time, mealtime, and bedtime using the toy provided by the program as well as other toys the child possessed.

#### 2.4.3. Treatment Fidelity 

The Music Together^®^ teacher used the structured curriculum and completed checklists to ensure they covered all aspects of the curriculum in each class. The music group leader was trained by an experienced Music Together^®^ director, and the play date group leader was trained by program staff experienced in running play dates. Sessions for both groups were recorded by research staff for observation to ensure adherence to the protocol. Using the Music Together weekly curriculum submitted each week by the teacher, research staff confirmed that all aspects of the curriculum were included in each class. For the play group, research staff viewed each class to confirm the facilitator only modeled play and refrained from providing feedback on developmental skills to the parents.

### 2.5. Measures

#### 2.5.1. Relative Reinforcing Value of Food (RRV) Task

Food/non-food reinforcement is a computerized task originally developed to assess the RRV_food_ in adults [41]. It has been adapted to assess children [42] and infants [43]. While in a highchair, research staff directed the infant’s attention to the task and completed familiarization, training, and data collection. During familiarization, the researcher showed the infant how to press the mouse button to hear a “funny noise”. The infant was then encouraged to press the button. Once the researcher determined that the infant understood how to control the noise with a button press, they transitioned to training where the infant practiced pressing the button to earn either a food or non-food reward. Once the researcher determined that the infant understood how to earn the reward, they transitioned to data collection. Throughout the experiment, researchers remained neutral in their instructions to the child and only used scripted cue phrases to engage with the infant.

When food was earned during the task, the researcher placed a piece of the food (approximately 1 cm × 1 cm × 1 cm) in front of them. Infants were given the opportunity to consume the food as it was earned. When music was earned, a 10 s song recording played while the researcher simultaneously played musical shakers. The songs used during assessments were not used in the music program to avoid familiarity bias. Infants worked for access to the food or non-food reinforcer in a counterbalanced order. The task (food or non-food) ended when the infant exhibited 1 of 4 behaviors consistently for 60 s (i.e., fussing/crying, communication, distraction, avoidance) to show that they were done playing. Between conditions (food/non-food), the researcher engaged the infant in 5 min of play. The schedule of reinforcement began with 1 button press to earn a reward and increased linearly every 2 trials, up to a maximum of 30 responses (i.e., 1, 1, 2, 2, 3, 3, …, 15, 15). The number of responses, number of rewards earned, and time spent in each session were recorded.

The dependent measure was a standard measure of reinforcing value, operationalized by P_max_, or the highest schedule the infant was required to complete to earn a food or non-food alternative. The food and non-food reinforcers were offered to the children in a sequential fashion in each cohort, and the food reinforcing ratio (FRR) was calculated as effort to obtain food out of total effort; however, for ease of interpretation and comparison with other populations, the term RRV_food_ is used instead of FRR throughout this article. The RRV_food_ was calculated as the proportion of responding for food relative to all responding [P_max_ food/(P_max_ food + P_max_ non-food alternative)]. The food stimulus in the task was a snack that infants liked and had consumed previously. Parents were asked to choose a snack that is ~4 kcal/g from 6 options: Gerber^®^ Lil’ Crunchies, Goldfish^®^ crackers, graham crackers, chocolate chip granola bars, animal crackers, and Keebler^®^ fudge-striped cookies. We considered providing a standardized food for all infants; however, it could be hazardous if a food was consumed for the first time in the lab due to potential allergies. Additionally, some infants may not eat a standardized food, which would make the task impossible to conduct. Thus, consistent with our prior work, we used the most preferred snack for each infant.

#### 2.5.2. Home Environmental Enrichment

##### Home Language Environment

Language data were collected using the Language Environment Analysis (LENA) device, a small “talk pedometer” recorder that fits in a pocket of a special vest worn by the child. At each timepoint, participants used LENA to collect 16 h recordings on two separate days. The LENA software suite (version 3.1.6) was used to generate the three basic quantitative estimates—adult word count, conversational turn count, and child vocalization count. For each variable, an average count from the two recording days was derived. The LENA system is widely used in both research and clinical settings [44,45,46]. 

##### Music @ Home Questionnaire

The Music @ Home Questionnaire was used to quantitatively assess the range of musical behaviors occurring in the home environment of families with young children [47]. The questionnaire includes 18 items scored on a 7-point agreement-disagreement scale. The total questionnaire score ranges from 18 to 126. The scale also comprises four subscales: (1) Parental beliefs about music (Cronbach’s α = 0.92), which consisted of four items pertaining to parents’ feelings about music; (2) Child engagement with music (Cronbach’s α = 0.65), which consisted of six items pertaining to child behaviors during music activities; (3) Parent initiation of singing (Cronbach’s α = 0.42), which consisted of five items about parents singing to their child; and (4) Parent initiation of music-making (Cronbach’s α = 0.90), which consisted of three items about parents’ use of music activities with their child. The confirmatory fit indices (CFI) showed moderate to good fit (CFI = 0.963) and high test–retest correlations (0.65 to 0.87) [47].

#### 2.5.3. Parent Satisfaction Questionnaire

We created a parent satisfaction questionnaire to gain insight on what parents thought about participating in the various research and weekly program components of the study. Parents ranked their satisfaction on a scale of 1 (not satisfied at all) to 5 (very satisfied) for multiple components related to research participation (i.e., the consent process) and multiple components of weekly program participation (i.e., class activities). Parents were also prompted to provide comments about their experience in the program. Survey responses generated a total satisfaction score, total research components score, and total weekly program component score. Parent comments were extracted for each prompt and grouped together by common themes. The questionnaire is available upon request.

### 2.6. Data Analysis

Group baseline characteristics were compared using ANOVA or Pearson’s chi-square test for frequency data. We performed an intention-to-treat analysis using all randomly assigned participants, including participants who dropped out of the program. The primary outcomes (RRV_food_ and home environmental enrichment measures) were analyzed with a mixed-model ANOVA, which handles missing data at random using maximum likelihood estimation and retains all randomly assigned subjects in the analysis [48]. In an effort to choose a reasonably fitted model satisfying the model assumptions, the residual plots and the residual-based fit statistics, such as Akaike’s Information Criterion, for various covariance structures were examined. The models included group, time (baseline, mid-, and post-intervention), and the group × time interaction as class variables using the unstructured (UN) covariance structure. As a sensitivity analysis, we also performed repeated measures ANOVA for those who had complete data for all timepoints (i.e., the completers) [49]. Effect sizes were reported as partial eta squared for mixed model ANOVA [50], and eta-squares were converted to Cohen’s f to ease model comparison. Effect sizes for repeated measures ANOVA similarly were computed from partial eta square [51] and converted to Cohen’s f using G*Power 3.1.9.6 [52]. Though *p*-values can inform whether an effect exists, Cohen’s f can help to assess the strength of that effect and contextualize the results. Given the small sample size, we focus on interpreting group differences when there are medium (0.25) to large (0.4) effect size estimates [53]. Since our study is a pilot study, knowing the expected effect size can inform power analyses for sample size determination of a future larger RCT. All models were performed using PROC MIXED in SAS version 9.4 (©2020, SAS Institute, Cary, NC, USA).

## 3. Results

### 3.1. Recruitment, Enrollment, and Randomization

A total of 178 individuals were approached in person at their child’s pediatrician appointment for eligibility screening. Of those who were approached, 85 individuals completed the screener for a 47.8% rate of in person screening. Individuals who declined to complete the screener cited the required time commitment, program location, and having a busy schedule as the reasons they were not interested. A total of 40 individuals also self-screened via a QR code posted to Facebook and on flyers distributed to community partners. In total, 125 individuals were screened for eligibility. Out of those who were screened, 77 individuals were eligible for participation (in person *n* = 58, 68.2%; self-screened *n* = 19, 47.5%). Of the 77 eligible individuals, 18 were enrolled for a total enrollment rate of 23.4% (in-person *n* = 7, 12.1%; self-screened *n* = 11; 57.9%). Before randomization, two families dropped out from the study; thus, we randomized a total of 16 families to either the music or play group. No significant differences were observed for baseline demographics of randomized participants, as shown in Table 1. Please refer to Figure 1 for the full recruitment and enrollment flow chart.

### 3.2. Attendance and Retention Rates

During the first 8-week semester, the rate of attendance was 59.38% (cohort 1 = 58.3%; cohort 2 = 60.7%). Participants attended an average of 4.75 (SD 3.42) classes during the first semester. During the second 8-week semester, the rate of attendance was 54.46% (cohort 1 = 57.1%; cohort 2 = 51.0%). Participants attended an average of 3.81 classes (SD 3.23) during the second semester. The mean percentage of classes attended over both semesters was slightly higher in the music group (54.36%, SD = 41.54%) as compared with the play group (51.32%, SD = 41.53%), though no significant difference was found (*p* = 0.885). The mean number of total classes attended was not significant between the groups [music group (8.63, SD = 6.59), play group (8.38, SD = 6.95), *p* = 0.942]. The retention rate for participating families at the mid-intervention assessment was 81.3% and at the post-intervention assessment was 50% (see Figure 2 for the full randomization and retention). There was no group difference in retention rate.

### 3.3. Relative Reinforcing Value of Food

**Figure 3:**
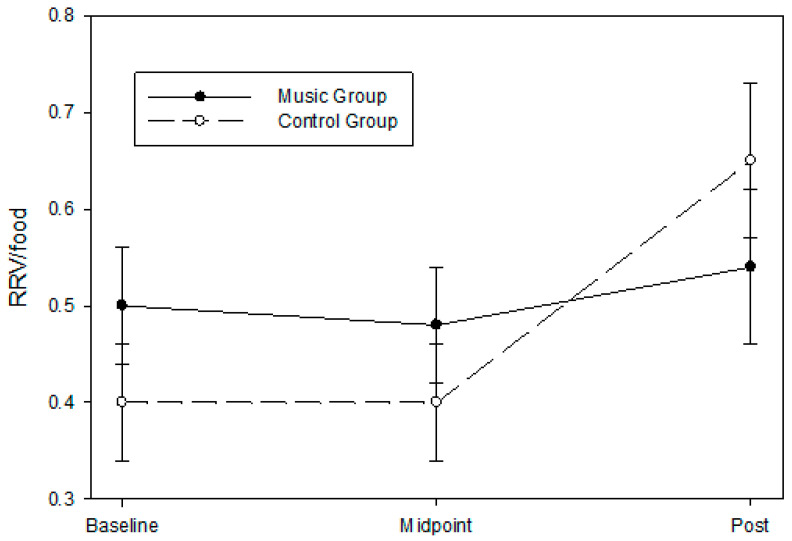
Changes in the RRV_food_ at all assessment timepoints (baseline, midpoint, and post-intervention) during the intervention. Sixteen 9- to 24-month-old infants were randomly assigned to either the music enrichment program (music group, *n* = 8) or play date control (control group, *n* = 8). A model adjusted for covariates is presented, and data are expressed as mean ± SEM. There were no significant differential group changes across time for the RRV_food_ (group × time; *p* = 0.206) for all randomized participants per intention-to-treat analysis, but the group difference corresponded to a moderate effect size (partial eta 2 = 0.032, Cohen’s f = 0.183).

There were no significant differential group changes across time for the RRV_food_ (group × time; *p* = 0.206) for all randomized participants per intention-to-treat analysis, but the group difference corresponded to a moderate effect size (partial eta ^2^ = 0.032, Cohen’s f = 0.183). Similarly, there was no significant group difference for the RRV_food_ for all completers (F = 1.47, *p* = 0.269), but the group difference corresponded to a large effect size (partial eta ^2^ = 0.0197, Cohen’s f = 0.495). Across time, all participants increased in their RRV_food_ (+0.143) from baseline to post-intervention (t = 2.54, *p* = 0.023). However, the music group had a lower degree of RRV_food_ increase than the control group from baseline to post-intervention (Figure 3). Please refer to Table 2 for descriptive statistics by timepoint and group for all randomized participants and Table 3 for all completers. We also included Appendix A Table A1 and Table A2 for the descriptive statistics of the differences between baseline and post-intervention within groups.

### 3.4. Home Environmental Enrichment Measures

#### 3.4.1. Home Language Environment Using LENA

##### Child Vocalizations (CV)

**Figure 4:**
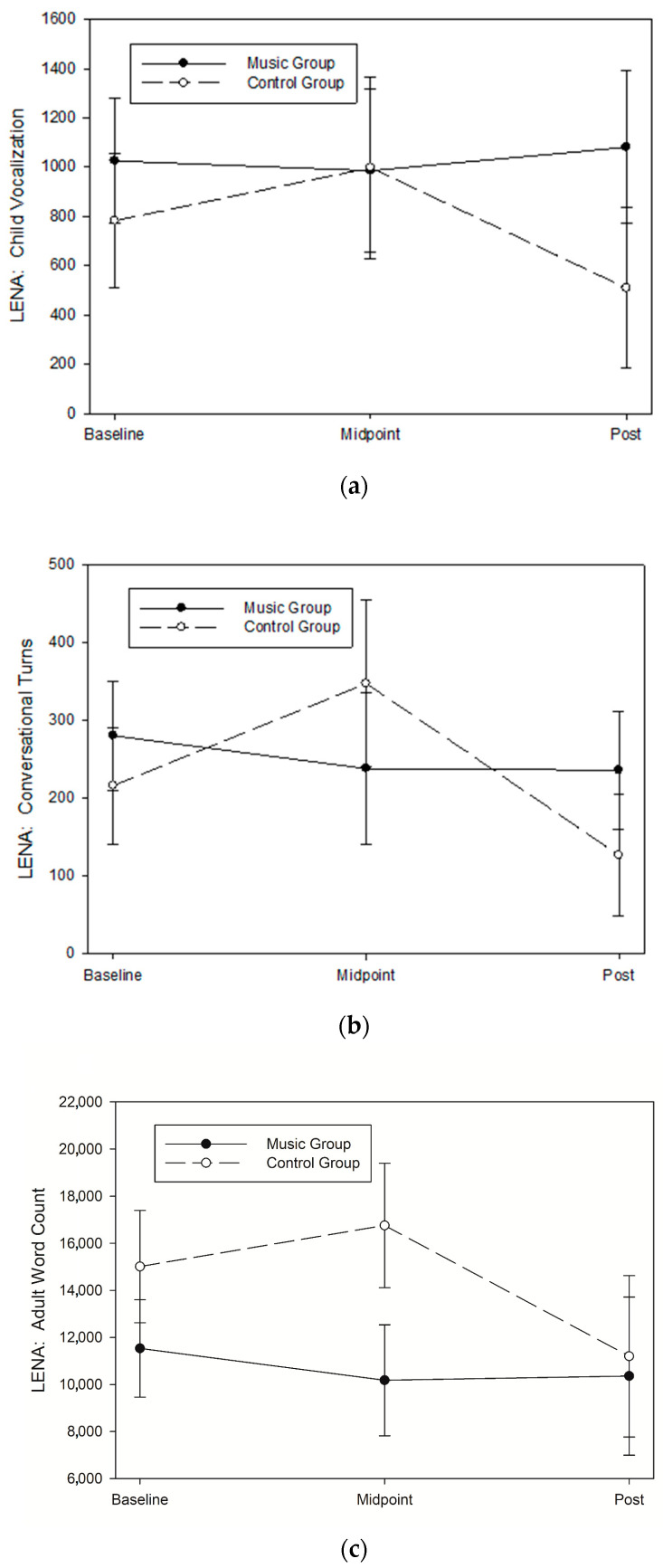
Changes in home language environment measured using Language Environment Analysis (LENA) device at all assessment timepoints (baseline, midpoint, and post-intervention) during the intervention. Sixteen 9- to 24-month-old infants were randomly assigned to either the music enrichment program (music group, *n* = 8) or play date control (control group, *n* = 8). A model adjusted for covariates is presented, and data are expressed as mean ± SEM. (**a**) There were no significant differential group changes across time for child vocalization (group × time; *p* = 0.366) for all randomized participants per intention-to-treat analysis, and the group difference corresponded to an effect size between small and medium (partial eta 2 = 0.020, Cohen’s f = 0.144). (**b**) There were no significant differential group changes across time for conversational turns (group × time; *p* = 0.340) for all randomized participants per intention-to-treat analysis, and the group difference corresponded to an effect size between small and medium (eta 2 = 0.021, Cohen’s f = 0.148). (**c**) There were no significant differential group changes across time for adult word count (group × time; *p* = 0.489) for all randomized participants per intention-to-treat analysis, and the group difference corresponded to a very small effect size (eta 2 = 0.012, Cohen’s f = 0.014).

There were no significant differential group changes across time for CV (group × time; *p* = 0.366) for all randomized participants per intention-to-treat analysis, and the group difference corresponded to an effect size between small and medium (partial eta ^2^ = 0.020, Cohen’s f = 0.144). Similarly, there was no significant group difference for CV for all completers (F = 1.68, *p* = 0.235), but the group difference corresponded to a large effect size (partial eta ^2^ = 0.252, Cohen’s f = 0.579). Though not significant, families in the music group did have a slight increase in CV from baseline to post-intervention compared to a decrease in CV for those in the control group (Figure 4a). Please refer to Table 2 for descriptive statistics by timepoint and group for all randomized participants and Table 3 for all completers. We also included Appendix A Table A1 and Table A2 for the descriptive statistics of the differences between baseline and post-intervention within groups.

##### Conversational Turns (CT)

There were no significant differential group changes across time for CT (group × time; *p* = 0.340) for all randomized participants per intention-to-treat analysis, and the group difference corresponded to an effect size between small and medium (eta ^2^ = 0.021, Cohen’s f = 0.148). Similarly, there was no significant group difference for CT for all completers (F = 1.54, *p* = 0.262), but the group difference corresponded to a large effect size (partial eta ^2^ = 0.235, Cohen’s f = 0.554). Though not significant, families in the music group did have an attenuated decrease in CT from baseline to post-intervention compared to a steeper decrease in CT for those in the control group (Figure 4b). Please refer to Table 2 for descriptive statistics by timepoint and group for all randomized participants and Table 3 for all completers. We also included Appendix A Table A1 and Table A2 for the descriptive statistics of the differences between baseline and post-intervention within groups.

##### Adult Word Count (AWC)

There were no significant differential group changes across time for AWC (group × time; *p* = 0.489) for all randomized participants per intention-to-treat analysis, and the group difference corresponded to a very small effect size (eta ^2^ = 0.012, Cohen’s f = 0.014). Similarly, there was no significant group difference for AWC for all completers (F = 1.67, *p* = 0.269), but the group difference corresponded to a large effect size (partial eta ^2^ = 0.251, Cohen’s f = 0.578). Though not significant, families in the music group did have an attenuated decrease in AWC from baseline to post-intervention compared to a steeper decrease in AWC for those in the control group (Figure 4c). Please refer to Table 2 for descriptive statistics by timepoint and group for all randomized participants and Table 3 for all completers. We also included Appendix A Table A1 and Table A2 for the descriptive statistics of the differences between baseline and post-intervention within groups.

#### 3.4.2. Music @ Home Questionnaire

**Figure 5:**
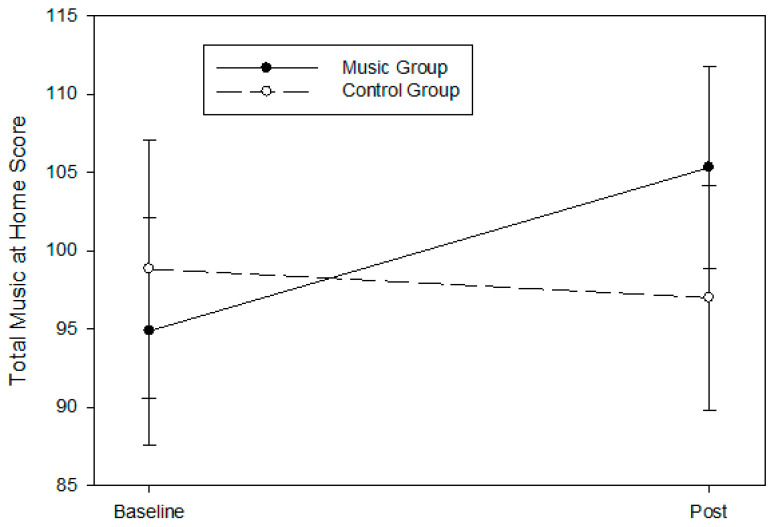
Changes in the total Music @ Home score at all assessment timepoints (baseline, and post-intervention) during the intervention. Sixteen 9- to 24-month-old infants were randomly assigned to either the music enrichment program (music group, *n* = 8) or play date control (control group, *n* = 8). A model adjusted for covariates is presented, and data are expressed as mean ± SEM. There were borderline group differences for the total scores on the Music @ Home questionnaire (group × time, *p* = 0.062; eta 2 = 0.074, Cohen’s f = 0.283) for all randomized participants per intention-to-treat analysis, and the group difference corresponded to a large effect size.

We observed borderline group differences for the total scores on the Music @ Home questionnaire from baseline to post-intervention (group × time, *p* = 0.062; eta ^2^ = 0.074, Cohen’s f = 0.283) for all randomized participants per intention-to-treat analysis, but no significant group difference for all completers (F = 3.83, *p* = 0.108, partial eta ^2^ = 0.433, Cohen’s f = 0.874) (Figure 5). The effect sizes for both analyses corresponded to large effect sizes. We also observed borderline group differences for the subscales of parent initiation of singing (group × time, p = 0.089; eta ^2^ = 0.061, Cohen’s f = 0.253) and parent initiation of music-making (group × time, p = 0.074; eta ^2^ = 0.068, Cohen’s f = 0.269) for all randomized participants per intention-to-treat analysis, but no significant group difference for all completers [parent initiation of singing (F = 3.03, p = 0.142, partial eta ^2^ = 0.378, Cohen’s f = 0.779) and parent initiation of music-making (F = 1.62, p = 0.259, partial eta ^2^ = 0.245, Cohen’s f = 0.570)]. Effects observed corresponded to a large size. Please refer to Table 2 for descriptive statistics by timepoint and group for all randomized participants and Table 3 for all completers. We also included Appendix A Table A1 and Table A2 for the descriptive statistics of the differences between baseline and post-intervention within groups.

### 3.5. Parent Satisfaction Questionnaire

Seven parents who completed the entire program completed the satisfaction questionnaire at the conclusion of the program. Overall, parents reported high satisfaction with all aspects of the program (Table 4). The mean score for overall satisfaction with the program was 4.86 (SD = 0.38; range = 2–5). Regarding research components of the study, the total mean score was 4.80 (SD = 0.50; range 3–5). Responses to the weekly program components yielded a mean score of 4.34 (SD = 0.83; range 2–5). When asked why they chose to participate in this program, parents commented, “to let my child experience something new,” and “help out with research and meet new people”. Parents said the most helpful aspects of the program were “having weekly one-on-one time with their child” and “social interaction for my child”. Regarding changes that parents made to their daily interactions because of attending the program, they said, “we now have separate music and play time,” and “we play and dance more often” (see Table 5 for complete answers).

## 4. Discussion

This pilot randomized controlled trial (RCT) tested whether a music enrichment program impacted the relative reinforcing value of food (RRV_food_) and home environmental enrichment. We also tested the feasibility of this type of program conducted among families from low-SES backgrounds and assessed parents’ impressions of the acceptability of the intervention. Our intervention was well-received by parents and demonstrated large effects on an increased use of music at home. We did not find significant group differences on the RRV_food_ and home language environment, but some of the effect sizes were medium-to-large. Due to a small sample size, the results of our intervention need to be interpreted with caution.

### 4.1. Intervention Effectiveness

It was expected that the music enrichment program would increase the reinforcing value of music, which would in turn decrease the overall RRV_food_ of participants in the music group. Though the group difference was not significant, there was a moderate to large effect of the music enrichment program on lowering the RRV_food_ increase as the infants aged. Future research with a larger sample is warranted, and incorporating a longer follow-up to examine the potential intervention impacts on feeding- and weight-related outcomes is needed. Reinforcing value of food is related to eating and energy intake and obesity [2,54,55]. Changes in the RRV_food_ should be related to changes in energy intake. Non-food alternatives may not change one’s homeostatic regulation of energy intake; however, changes in hedonic eating behavior, which is thought to be a significant source of excess caloric intake [56], should decrease when alternative reinforcers are strengthened. This demonstrates that healthy eating does not need to be targeted if complimentary or substitute behaviors can be identified and manipulated.

Our results also indicated some signals for the effect of music enrichment on the home language environment assessed using LENA. There were increases in child vocalization, conversational turns, and adult word count post-intervention for families who were in the music group compared to decreases in the control group. This is best represented by one parent in the music group who said, “My daughter has learned the words to more songs and the actions as well”. The language environment plays a critical role in language acquisition. The quality of the early home language environment, and conversational turns in particular, is a strong predictor of a child’s language development [57]. Research of music interventions has shown that engaging in music activities increases parent–child mutual attunement, a behavior that is also required for conversational turns [58]. Research regarding the role of music enrichment programs to enhance the overall language environment is limited. However, it may be that an increased quality of parent–child interaction during music engagement and boosted use of music at home leads to a richer language environment and thus an enriched home environment. In one study using the Music@Home questionnaire, more parental singing was related to better word comprehension in 12-month-old infants, and a higher total score was associated with higher gestural communication scores [59]. Additional research investigating the association between the use of music at home and the language environment is needed.

The most promising results of this study are the large impacts on parental reports of music used at home post-intervention for families in the music group. There was a medium-to-large effect size of the intervention on the total music at home score. When we examined the subscales separately, parents in the music group reported an increase in initiation of singing and music-making at home. These effects were all medium-to-large in size. This aligned with the survey response of one parent in the music group, in which she indicated that they are now using homemade instruments more often at home after participating in the music program. Interestingly, we received feedback that parents in the play group also played and danced more often with their child at home after the play date program. Parents singing to their children is known to decrease as children become older [32,37]. Yet, in our sample, parents in the music group indicated they initiated more singing and music-making at home at the conclusion of the 16-week intervention. Participating in the music enrichment program may have helped parents transfer the skills they learned in the classroom to home. This might also suggest that parents practice the skills they have learned at home. Caregiver singing provides many benefits, such as sustained infant attention and dyad regulation, which are known for better health and developmental outcomes [32,33,60]. Parents of low-SES backgrounds face additional disparities such as poor parental mental health, high parental stress, and lack of access to resources that can negatively impact the quality of the home environment [61]. Therefore, increasing the use of music at home by families in our study may be important at supporting overall child health and development.

It is important to note that, our play date control might have unintentionally provided social interactions that might have enriched the home environment of those in the control group. Social reinforcers can be powerful reinforcers, as children value positive interactions with adults, especially with parents during infancy, because they are their primary source of social interaction and support [62,63]. Pleasant parental interactions, such as playtime, could be pleasurable and enriching. Studies have shown that high-quality parent–infant interactions during playtime were associated with a lower weight status cross-sectionally [64] and normal weight gain trajectories longitudinally [65]. In the future, perhaps a different type of educational program can serve as the active control group in our study design.

### 4.2. Implementation Data-Feasibility and Acceptability

We recruited our families from a hospital-affiliated primary care clinic that primarily serves diverse, low-income populations. Families of children between 9 and 24 months were identified via electronic medical records. They were approached to complete an eligibility screener at their scheduled well-child appointment. After learning about the study, approximately 50% of families refused the screener due to being too busy for the time commitment of weekly classes, location, or schedule conflicts. In addition to in person screening, we also had families who self-screened through flyers distributed to local non-profit organizations and community partners who serve families of low-SES backgrounds, as well as via our hospital’s social media through a Facebook post. From our recruitment record, we found that the enrollment percentage from our self-screened method was higher (57.9%) than from our in-person approach at the clinic (12.1%). This might reiterate the importance of collaborating/working with community partners who have established relationships with families of interest in the community. Many individuals self-screened from Facebook in our study did not qualify for the study because they were mainly mid–upper-income families who follow our hospital Facebook page. Social media platforms, however, could be a great means to connect with families of interest to promote research studies, especially with diverse, low-income users who seek to serve a common cause. For each cohort of the study, our recruitment effort was in a span of 9 weeks because we enrolled and assessed all eligible families 2 weeks prior to the start of the program. As a result, we lost many eligible families during the waiting period. In the future, we might need to build in activities that allow us to engage with families who are eligible for the study during the waiting period for enrollment.

For the present study, the class attendance rate was about 60%, which is considered high compared to previous intervention research among families of low-SES backgrounds who have young children [66,67,68]. In addition, previous research has considered 60% of class attendance as sufficient for families to receive an impact from the intervention [35]. This minimum attendance threshold was achieved by 50% of the families in our study. The class attendance rate of our study is higher than other intervention research conducted among this population, which could be due to the several critical steps taken to ensure that participation in the program did not create additional burden/stress to the families. These approaches were implemented after careful consideration of the literature [69,70,71,72,73] and advice received from community partners who regularly work with families experiencing similar barriers. In our study, we provided dinner each week to all families before the start of the program. We also provided childcare if the participating parent brought siblings or other family members to the sessions. All participants were offered free transportation through Lyft service to attend classes if they did not have reliable transportation. Lastly, they also received USD 20 each week for participation, which was intended to offset any costs related to their participation (i.e., gas for the car, childcare, etc.). In terms of retention rate, we had a greater retention rate at the 8-week mid-intervention timepoint than at the 16-week post-intervention (81.3% vs. 50%). Some families dropped out between weeks 8 and 16 due to family circumstances such as separation of parents and changes in child custody. Early intervention and prevention efforts targeting high-risk households can be difficult to implement due to multiple social risk factors. Potentially, programs that are shorter in duration but are more targeted with higher “dosage” of the treatment might work better for this population. In addition, we might have missed the opportunity by not scheduling families right away at their last class to attend their post-intervention assessment. The location of the assessment is different from the location of their classes, which might have caused the dropout. In the future, we might consider having assessments at the same location.

Overall, the intervention was well-liked by families who completed the music and play date groups. The mean enjoyment score for each item in both categories—research process and weekly program—was above 4.5 (5 as the maximum). The lowest score we received that was below average was the weekly meals during intervention. In the open-ended portion of the satisfaction questionnaire, one participant suggested having more options for the meals served weekly. Since the present study is a pilot RCT, to be cost-effective and to reduce staff burden, we rotated 3–4 meals (i.e., lasagna, tacos, pizzas, and hotdogs with various side dishes) throughout the intervention period. Those meals were suggested by families in cohort 1 at the beginning of the program, which we continued to serve to families in cohort 2. In the future, we could consider surveying families from each cohort at the beginning and midpoint of the program for meal suggestions.

Two themes emerged when families were asked why they chose to participate in the program. They believed our program would benefit their child/family and they wanted to contribute to research. These findings are consistent with previous research in that one of the major determining factors for families to participate in research studies is for the potential gain of their family member (i.e., child/ren) who is involved [74] and for the greater good of society [75,76]. Furthermore, two themes also emerged when families were asked what they found most helpful with the program. They valued the increase in parent–child and social interactions their child experienced through the program, which reiterates the benefit of group-based versus individual-based programming [74]. This might be an important factor to consider when designing a program for diverse, low-income families with young children. Families also found the components of our program to be helpful to their participation. One mentioned that they liked that childcare was provided, and another family complimented the research team’s effective communication throughout the duration of their participation. Families were able to reach a staff member via email, text message, or phone call, and for consistency and rapport, the same staff member responded to the same families each time. Staff members were typically able to respond to participant communication within 24 h.

### 4.3. Limitations

The small sample size is a primary limitation of this pilot RCT. As a pilot study, we did not power this study to detect effects on the RRV_food_ and home environment enrichment measures. Instead, our larger goal for this study was feasibility and acceptability. As such, our findings should be interpreted with caution and replicated in a larger sample. Our small sample size also limited our ability to examine moderating effects of child and parent characteristics on intervention effects. While we recruited children from families of low-SES, some of our participants had a relatively low RRV_food_ at baseline, leaving little room for improvement. Music enrichment programs may have stronger impacts in infants with a higher RRV_food_ at baseline who might have a higher obesity risk in addition to the SES risk. Besides, we did not collect dietary intake data in this study, so we are not able to examine if increasing music reinforcement helps to decrease food reinforcement. It was our hypothesis that by increasing the reinforcing value of music, children will find pleasure in music-making and thus decrease their motivation for food. The Music @ Home questionnaire is a self-reported questionnaire, which could lead to parental response bias, especially those in the music group. It is important to point out that two of our subscales, child engagement with music and parent initiation of singing of the Music @ Home questionnaire, had a Cronbach’s α reliability of less than 0.7. This could be due to our small sample size. Lastly, only families who completed the study responded to the parent satisfaction questionnaire, which might cause biases to the results. We sent the questionnaire to all 18 families who were enrolled in hope of obtaining their feedback for any further reasons as to why they dropped out of the study. It would have been beneficial to gain insight from participants who did not complete the program to further refine our intervention.

### 4.4. Conclusions/Future Directions

Taken together, the results of this pilot RCT suggest that a community-based music enrichment program is feasible and acceptable to families of low-SES backgrounds. Parent feedback indicated the intervention was well-liked, and steps we took to help reduce barriers to participation were key to these families. Overall, we found some intervention effects on the RRV_food_ and some of the home environmental enrichment measures. Early intervention programs entail a huge commitment of time and energy by parents, particularly mothers. Family members in low-SES households often face many life challenges that preclude their participation. Adverse social determinants of health, also called social risk factors (SRFs), influence the outcomes of many early childhood intervention and prevention programs and are often caused by structural inequities and historical and current limitations in access and resources [77]. Two studies, one in a safety net pediatric practice and one in a hospital, found that up to 83% of families had one or more basic need that was not met (employment 52%; education 34%; childcare 19%; food insecurity 16%; housing 10%) [16,17]. These SRFs are often associated with practical barriers to program participation, including lack of transportation, erratic and unpredictable work hours, lack of childcare, and food insecurity. Basic needs must take priority. While families with low incomes are generally motivated to make changes to improve their health, they often lack the resources to do so [14,15]. Additionally, dealing with social risk factors can tax people’s physical, emotional, and mental energy and add to their cognitive load, preventing them from participating or continuing in programs [78,79]. An overloaded working memory (high cognitive load) leads directly to difficulty learning [80], poor time management [81], and inappropriate or unresponsive parenting [82]. Therefore, addressing social risk factors prior to or concurrently with participating in early intervention or prevention programs such as a music enrichment program may promote better enrollment and completion rates. In conclusion, future research is warranted, as music enrichment programs may be a high-impact, low-cost strategy to address socioeconomic disparities, as they could address multiple outcomes (i.e., reduce motivation to eat, increase language outcomes) with a single intervention.

## Figures and Tables

**Table 1:** Participant Demographic Characteristics.

Variable	Music Group (*n* = 8)	Control Group (*n* = 8)
Child		
Sex, male	4 (50.0)	4 (50.0)
Age, mo	15.84 ± 4.84 (9.76, 22.95)	17.11 ± 4.73 (9.24, 24.89)
Race		
Black	3 (37.5)	7 (87.5)
White	3 (37.5)	0 (0)
Multiracial	2 (25.0)	1 (12.5)
Ethnicity		
Hispanic	1 (12.5)	1 (12.5)
Birth weight, kg	3.36 ± 0.21 (3.04, 3.63)	3.14 ± 0.69 (2.31, 4.45)
Mother		
Age, y	30.43 ± 5.02 (23, 37)	33.25 ± 5.06 (25, 42)
Race		
Black	3 (37.5)	6 (75.0)
White	4 (50.0)	1 (12.5)
Multiracial	1 (12.5)	1 (12.5)
Ethnicity		
Hispanic	1 (12.5)	0 (0)
Marital status		
Single	3 (37.5)	4 (50.0)
Education Level		
Some college or less	4 (50.0)	7 (87.5)
College graduate or more	4 (50.0)	1 (12.5)
Current BMI, kg/m^2^	33.61 ± 7.42 (25.55, 43.72)	36.31 ± 9.09 (21.35, 50.35)
Normal	0 (0)	1 (14.3)
Overweight	4 (50.0)	0 (0)
Obese	4(50.0)	6 (85.7)
Household		
Family members in the household	5.00 ± 1.85 (3, 8)	4.75 ± 1.16 (3, 6)
Number of children	3.00 ± 2.00 (0, 5)	3.25 ± 1.28 (1, 5)
Family Annual Income, USD	18,745.50 ± 12,222.62 (6000, 40,800)	27,474.00 ± 8986.13 (12,000, 36,000)
Poverty Status		
Above poverty threshold	1 (12.5)	1 (12.5)
Below poverty threshold	7 (87.5)	7 (87.5)

**Table 2:** Descriptive statistics by timepoint and group per intention-to-treat analysis (*n* = 16).

					Music Group						Control Group					
					Baseline		Mid		Post		Baseline		Mid		Post	
	F	*p*-Value	Partial eta^2^	Cohen’s f	Mean	SEM	Mean	SEM	Mean	SEM	Mean	SEM	Mean	SEM	Mean	SEM
Proximal obesity measure																
RRVfood	1.77	0.21	0.03	0.18	0.50	0.06	0.48	0.06	0.54	0.08	0.40	0.06	0.40	0.06	0.65	0.08
LENA																
child vocalization	1.09	0.37	0.02	0.14	1025.38	254.09	985.65	331.86	1080.73	310.22	782.71	271.63	997.77	368.64	509.22	325.35
conversational turns	1.17	0.34	0.02	0.15	280.19	69.99	238.00	97.61	235.90	75.63	216.07	74.82	347.08	107.01	126.66	77.87
adult word count	0.76	0.49	0.01	0.12	11,535.00	2065.31	10,182.00	2359.50	10,357.00	3358.62	15,013.00	2384.81	16,758.00	2647.33	11,198.00	3431.70
Music @ Home Questionnaire																
parental beliefs aboutmusic	0.67	0.43	0.13	0.11	19.97	2.03	N/A	N/A	22.32	1.95	22.17	2.29	N/A	N/A	22.69	2.09
child engagement withmusic	0.00	0.98	0.00	0.00	36.56	2.51	N/A	N/A	36.93	3.08	33.50	2.84	N/A	N/A	33.79	3.33
parent initiation ofsinging	3.41	0.09	0.06	0.25	24.78	2.13	N/A	N/A	28.22	1.86	27.67	2.34	N/A	N/A	25.27	1.90
parent initiation of music-making	3.84	0.07	0.07	0.27	13.60	1.96	N/A	N/A	16.74	0.89	16.33	2.21	N/A	N/A	15.27	0.96
total music @ home score	4.25	0.06	0.06	0.28	94.87	7.25	N/A	N/A	105.31	6.49	98.83	8.26	N/A	N/A	96.99	7.15

**Table 3:** Descriptive statistics by timepoint and group per repeated measured ANOVA.

					Music Group						Control Group					
					Baseline		Mid		Post		Baseline		Mid		Post	
	F	*p*-Value	Partial eta^2^	Cohen’s f	Mean	SEM	Mean	SEM	Mean	SEM	Mean	SEM	Mean	SEM	Mean	SEM
Proximal obesity measure																
RRVfood (*n* = 8)	1.47	0.27	0.02	0.50	0.48	0.08	0.50	0.07	0.53	0.08	0.35	0.08	0.40	0.07	0.62	0.08
LENA																
child vocalization (*n* = 8)	1.68	0.24	0.25	0.28	628.17	263.89	832.33	438.96	924.00	358.58	1086.88	228.54	813.25	380.15	576.75	310.54
conversational turns (*n* = 8)	1.54	0.26	0.24	0.55	154.83	49.62	128.17	65.95	168.33	77.05	285.13	42.97	248.88	57.11	149.13	66.73
adult word count (*n* = 8)	1.67	0.24	0.25	0.58	8392.67	1625.78	6116.33	1867.00	9974.50	4227.72	13,535.00	1407.96	13,410.88	1616.87	10,913.88	3661.32
Music @ Home Questionnaire																
parental beliefs about music (*n* = 7)	0.91	0.38	0.15	0.43	19.33	4.25	N/A	N/A	22.33	3.38	22.25	3.68	N/A	N/A	22.75	2.93
child engagement with music (*n* = 7)	0.02	0.90	0.00	0.06	37.33	3.89	N/A	N/A	38.00	4.46	34.25	3.37	N/A	N/A	34.50	3.87
parent initiation of singing (*n* = 7)	3.03	0.14	0.38	0.78	24.00	4.28	N/A	N/A	28.67	2.60	28.25	3.71	N/A	N/A	25.50	2.25
parent initiation of music-making (*n* = 7)	1.62	0.26	0.25	0.57	14.33	3.82	N/A	N/A	17.33	1.36	17.00	3.31	N/A	N/A	15.50	1.18
total music @ home score (*n* = 7)	3.83	0.11	0.43	0.87	95.00	14.00	N/A	N/A	106.33	11.33	100.50	12.13	N/A	N/A	98.25	9.81

**Table 4:** Parent satisfaction responses.

Questionnaire Items	Mean (SD)	Range
Research process items		
Enrollment/consent process	4.86 (0.38)	4–5
Interactions with staff members	4.86 (0.38)	4–5
Communication (emails, text messages, phone calls)	4.86 (0.38)	4–5
Home visits	4.71 (0.76)	3–5
Research assessments (food game, playing with/feeding your child, language recorder)	4.86 (0.38)	4–5
Filling out questionnaires	4.57 (0.79)	3–5
Participant payments	4.86 (0.38)	4–5
Weekly class items		
Location of weekly classes	4.71 (0.76)	3–5
Transportation (if used)	4 (1.41)	3–5
Weekly meals	4.43 (1.13)	2–5
Weekly class activities	4.71 (0.76)	3–5
Take home materials	4.86 (0.38)	4–5

**Table 5:** Parent comments on satisfaction questionnaire prompts.

Prompt	Themes	Individual Comments	
		Music Group	Play Group
Why did you decide to participate in this program?	Child/family benefits	“for my son to participate in a music class and it was paid”	“It was very helpful for my child”
		“to let my child experience something new and interact with other children”	
		“To get more information on things that will help benefit my family”	
		“we kinda used it like a weekly play date”	
	Research contribution		“Help out with Research, meet new people, a place for my child to play”
			“Research studies are important process improvements”
What did you find most helpful?	Parent–child or social interaction	“social interaction”	“My child playing with others and the care they showed me and my child”
		“interaction with other kids his age”	
		“It helped me and my daughter become a little closer and bond over music”	
		“We made some good friends. I loved the weekly one on one time with my child.	
	Program components	“Music”	“Having childcare for my other kid. It made it helpful to focus on what we came to do”
			“Communication during this study was excellent”
What suggestions do you have for improving the program for other families?	Weekly meals		“more food options”
	Continue the program	“keep it going! There should be more activities for parents and children to do together”	
Please describe any changes in how you play or use music with your child at home due to participating in this program.	None	“it’s hasnt changed”	
	Increased play/music	“we don’t have as many instruments (as used in class)”	“We have separate music time and play time”
		“I have used homemade instruments more often”	“We play and dance more often”
			“He loves to play more now and dance too with others”
	Learning	“My daughter has learned the words to more songs and the actions as well”	

## Data Availability

The datasets from this study are available from the corresponding author upon request.

## Appendix A

**Table A1:** Descriptive statistics of the differences between baseline and post-intervention within groups per intention-to-treat analysis (n = 16).

				Music Group							Control Group			
				Baseline		Post					Baseline		Post	
	t	*p*-Value	Cohen’s f	Mean	SEM	Mean	SEM	t	*p*-Value	Cohen’s f	Mean	SEM	Mean	SEM
Proximal obesity measure														
RRVfood	0.490	0.631	0.101	0.496	0.062	0.535	0.079	3.100	0.008	0.631	0.400	0.062	0.647	0.080
LENA														
child vocalization	−0.420	0.679	0.035	1025.380	254.090	1080.730	310.220	−0.260	0.801	0.162	782.710	271.630	509.220	325.350
conversational turns	−0.670	0.513	0.108	280.190	69.986	235.900	75.630	−1.330	0.205	0.207	216.070	74.820	126.660	77.868
adult word count	−0.360	0.726	0.077	11,535.000	2065.310	10,357.000	3358.620	−1.150	0.274	0.232	15,013.000	2384.810	11,198.000	3431.700
Music @ Home Questionnaire														
parental beliefs about music	1.430	0.178	0.209	19.969	2.033	22.323	1.953	0.350	0.733	0.042	22.167	2.291	22.692	2.089
child engagement with music	0.170	0.870	0.023	36.563	2.509	36.928	3.083	0.150	0.886	0.017	33.500	2.839	33.788	3.331
parent initiation of singing	1.520	0.155	0.305	24.778	2.132	28.224	1.864	−1.090	0.299	0.200	27.667	2.344	25.274	1.902
parent initiation of music-making	2.120	0.055	0.390	13.595	1.956	16.737	0.889	−0.680	0.507	0.118	16.333	2.211	15.269	0.958
total music @ home score	2.370	0.036	0.269	94.873	7.250	105.310	6.489	−0.460	0.654	0.042	98.833	8.258	96.989	7.145

**Table A2:** Descriptive statistics of the differences between baseline and post-intervention within groups per repeated measured ANOVA.

				Music Group							Control Group			
				Baseline		Post					Baseline		Post	
	F	*p*-Value	Cohen’s f	Mean	SEM	Mean	SEM	F	*p*-Value	Cohen’s f	Mean	SEM	Mean	SEM
Proximal obesity measure														
RRVfood (n = 8)	1.150	0.362	0.180	0.475	0.079	0.525	0.084	6.280	0.087	0.844	0.350	0.079	0.623	0.084
LENA														
child vocalization (n = 8)	1.140	0.364	0.274	628.167	263.892	924.000	358.575	2.520	0.210	0.473	1086.875	228.537	576.750	310.535
conversational turns (n = 8)	0.060	0.818	0.062	154.833	49.623	168.333	77.055	2.630	0.204	0.620	285.125	42.975	149.125	66.731
adult word count (n = 8)	0.170	0.706	0.156	8392.667	1625.777	9974.500	4227.723	1.390	0.324	0.259	13,535.000	1407.964	10,913.880	3661.315
Music @ Home Questionnaire														
parental beliefs about music (n = 7)	1.290	0.375	0.227	19.333	4.254	22.333	3.380	0.180	0.703	0.038	22.250	3.684	22.750	2.928
child engagement with music (n = 7)	0.040	0.868	0.046	37.333	3.894	38.000	4.465	0.160	0.718	0.017	34.250	3.372	34.500	3.867
parent initiation of singing (n = 7)	1.000	0.423	0.392	24.000	4.280	28.667	2.603	3.670	0.151	0.231	28.250	3.706	25.500	2.255
parent initiation of music-making (n = 7)	0.690	0.493	0.334	14.333	3.818	17.333	1.358	0.930	0.406	0.167	17.000	3.307	15.500	1.176
total music @ home score (n = 7)	2.980	0.227	0.258	95.000	14.002	106.333	11.330	0.390	0.575	0.051	100.500	12.126	98.250	9.812
